# Prioritizing mental health through humanitarian, peacebuilding and development actions

**DOI:** 10.2471/BLT.21.287714

**Published:** 2022-03-01

**Authors:** Ren Minghui, Feng Zhao

**Affiliations:** a Universal Health Coverage / Communicable and Noncommunicable Diseases Division, World Health Organization, Avenue Appia 20, 1211 Geneva 27, Switzerland.; b World Bank Group, Washington, DC, United States of America.

The coronavirus disease 2019 (COVID-19) pandemic is deeply affecting people’s mental health. Estimates indicate the pandemic has led to at least a 25% increase in the prevalence of common conditions such as depression and anxiety disorders, with women and young people most affected.^1^

Mental health was a largely unaddressed challenge before the pandemic. Mental health conditions are among the leading causes of disability worldwide, with a cumulative global loss of economic output estimated to exceed 16 trillion United States dollars between 2011 and 2030.^2^ Yet despite the availability of cost-effective and evidence-based strategies and interventions, attention to and investment in mental health have been limited.^3^ Before the pandemic, governments in low- and middle-income countries were typically allocating less than 2% of their health budgets to mental health and studies suggest that more than 70% of people with the severe condition of psychosis did not receive any care.^4^

The COVID-19 pandemic has exposed the consequences and limitations of underinvestment in mental health services. In a World Health Organization (WHO) survey on the impact of the pandemic on essential health services, only 17% (20/116) of WHO Member States who reported integrating mental health and psychosocial support within their COVID-19 response plans also reported allocating full funding to these components.^5^

Emergencies that exacerbate mental health challenges – such as the COVID-19 pandemic – can and do bring renewed attention to the mental health and well-being of those affected.^6^ Governments endorsed the importance of developing and strengthening action on mental health at the United Nations General Assembly in September 2020 and at the Seventy-fourth World Health Assembly in May 2021.^7^^,^^8^

WHO, the World Bank, Member States and partners have responded to the mental health and social needs brought about by the pandemic. WHO and Inter-Agency Standing Committee partners have provided technical support and guidance to countries in developing and coordinating effective mental health and psychosocial responses to the pandemic and have developed widely translated and adapted resources.^9^ The World Bank has taken steps to support mental health during the pandemic, such as through the Multiphase Programmatic Approach of its COVID-19 Strategic Preparedness and Response Program. This programme has enabled several of the 25 beneficiary countries to implement key mental health interventions at scale as part of the COVID-19 response.

Despite these efforts, more needs to be done to transform initial attention into sustained action. To ensure such comprehensive transition, we suggest putting forward three key actions. 

First, the global health and development community must identify mental health as a cross-cutting priority for humanitarian, peacebuilding and development actors, both in terms of sustainable recovery from the COVID-19 pandemic and of sustainable development. Mental health needs are often only recognized in the short-term, yet the mental health consequences of exposure to severe stress are long-term.^8^ Sustainable and comprehensive mental health systems require an approach that supports and enables governments to include and embed mental health across sectors and policy areas.

Second, we must reformulate health financing policies and essential packages of care, in line with the principles of universal health coverage and available evidence. Mental health conditions are rarely given a level of priority or resourcing that is proportionate to their public health and socioeconomic effects. Many of the tools and strategies necessary to build robust, integrated and resilient mental health systems are well established^10^ and have a demonstrable impact when accompanied by active engagement, commitment and investment. 

Third, we must progressively integrate mental health into health system strengthening efforts and existing financing instruments for health improvement and social development. Many innovations for delivering mental health interventions at community, primary health care and clinical care levels exist, providing practical solutions to constraints such as stigma and lack of a well-trained workforce. Such integration will require support from the existing financing instruments. Mental health can also be prioritized through mechanisms such as the World Bank’s health sector financing. Recently established mechanisms, notably the Multi-Partner Trust Fund to Catalyse Country Action for Noncommunicable Diseases and Mental Health, can generate domestic development, financing and advancement of mental health systems through cost-effective investment in proven approaches. The Global Financing Facility for Women, Children and Adolescents could also play an important role in supporting these population groups.

Only with consolidated action can mental health become a sustainable development priority.^11^ A broad coalition of key players at all levels is needed to move forward the mental health agenda. Focusing on responding to and recovering from the current crisis is insufficient. We must use a comprehensive approach to develop strong and resilient mental health systems capable of providing care and support for those who need it.
